# Advances in understanding Ton and Tol system motor proteins

**DOI:** 10.1042/BST20253128

**Published:** 2026-01-28

**Authors:** Herve Celia, Susan K. Buchanan, Istvan Botos

**Affiliations:** 1 Laboratory of Molecular Biology, National Institute of Diabetes & Digestive & Kidney Diseases, National Institutes of Health, Bethesda, MD, U.S.A.

**Keywords:** membrane proteins, molecular motors, transmembrane proteins

## Abstract

The Ton and Tol-Pal systems are molecular machines that are essential for survival of Gram-negative bacteria.Both use the energy derived from the proton gradient at the inner membrane to generate force on protein components at the outer membrane. Ton and Tol share extensive homology, but they fulfill different functions: Ton is involved in the active transport of essential nutrients from the extracellular media into the cell, while Tol maintains the outer membrane integrity and participates in the cell division process. Despite decades of biochemical and biophysical studies, the molecular mechanism coupling the proton gradient at the inner membrane with the propagation of force and movement to the outer membrane is not understood. In this review, we discuss the recent high-resolution structures obtained for both systems, and how these structures fit with existing mechanistic models.

## Introduction

The cell envelope of Gram-negative bacteria is composed of a double lipidic membrane that confers an efficient barrier to external stress [1]. The inner (IM) and outer (OM) membranes are separated by a 15–40nm wide periplasmic space, which contains an extended rigid network of polymerized peptidoglycan (PG), the cell wall, that modulates the shape of the bacterial cell. The periplasmic space is devoid of an energy source; therefore, any active process taking place at the OM relies on long-distance transfer of energy produced in the IM or cytoplasm. The Ton and Tol-Pal systems use the proton motive force (pmf) at the IM to energize processes at the OM. The generated force is propagated across the periplasm by specialized elongated proteins that interact with protein partners at the OM.

**Figure 1:**
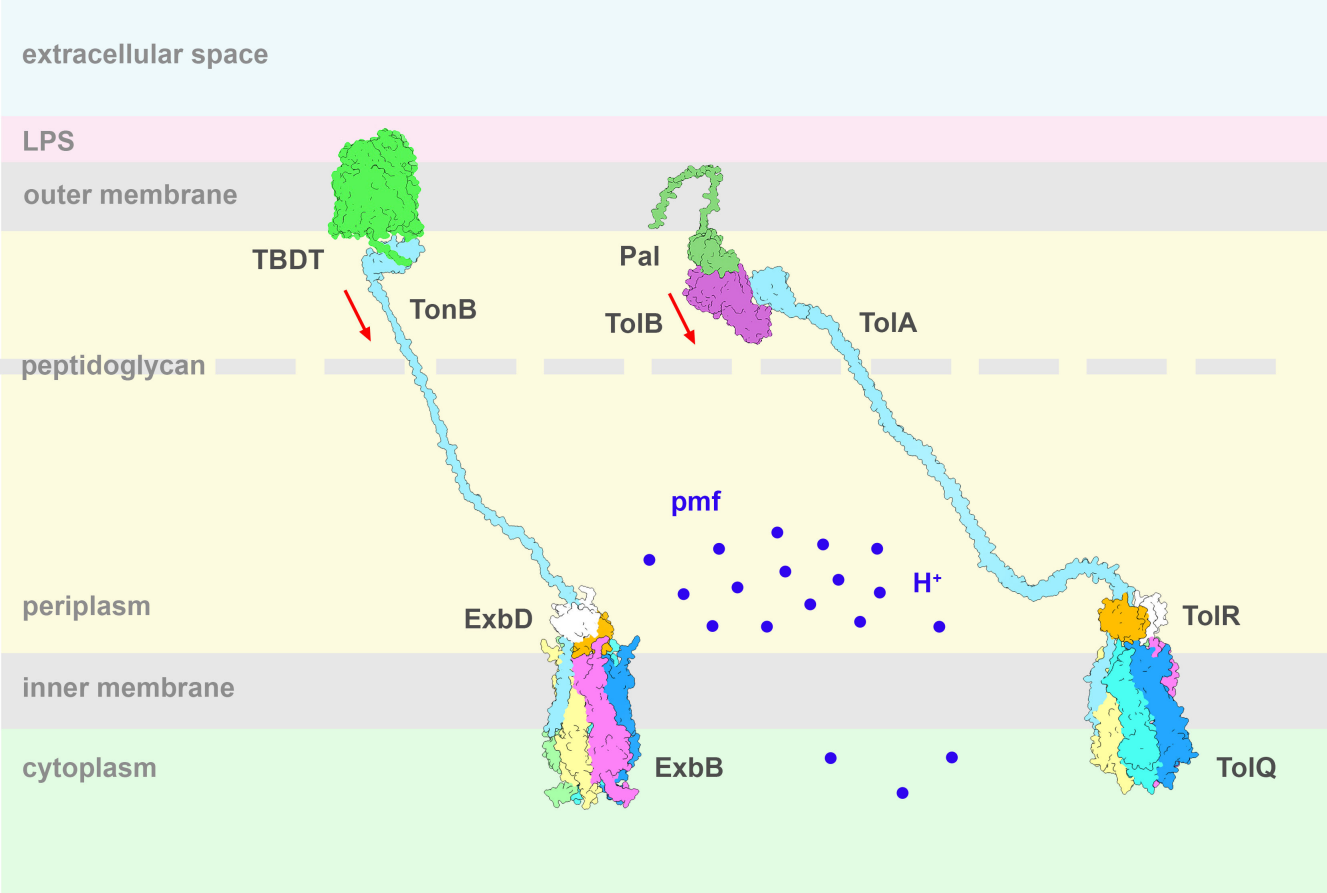
Schematic of the Ton and Tol systems. In the IM five ExbB molecules (dark blue, pink, yellow, green, and teal) form a pentamer with a hydrophobic pore that harbors an ExbD dimer (white and orange). TonB (light blue) has a transmembrane helix, an elongated periplasmic linker region, and a C-terminal domain. The C-terminal domain crosses the PG layer and interacts with TonB-dependent transporters (TBDT, green) in the OM. The ExbBD complex translocates protons from the periplasm to the cytoplasm, generating force that is transmitted by TonB to open a channel in the TBDT, allowing nutrients to diffuse into the periplasmic space. The Tol system has a similar architecture: a pentameric TolQ surrounding a TolQ dimer in a hydrophobic pore. The TolA TM connects through the periplasmic space to a compact C-terminal domain located outside the peptidoglycan and interacts with the TolB-Pal (green and purple) complex at the outer membrane. TolQR also uses the pmf as an energy source. The force is transmitted by TolA to the OM where it disrupts the TolB-Pal complex, letting Pal interact with the PG. The red arrows show the direction of the pulling force applied to the TBDT or TolB-Pal at the OM.

Ton is involved in the uptake of nutrients essential for bacterial survival such as iron siderophores, vitamin B12, zinc, glycans, and peptides [2]. At the IM, the ExbB and ExbD subunits associate to form part of the motor complex. The translocation of protons through the ExbBD subcomplex presumably generates a power stroke that is transmitted to the TonB subunit. TonB then transfers force through its elongated periplasmic domain to specific transporters in the OM, resulting in the active transport of bound nutrients into the periplasmic space [3,4] (Figure 1).

The Tol-Pal system is involved in the cell division process and maintenance of the OM integrity and is also thought to play a role in lipid homeostasis and import of chromate and phosphate [5–8]. As for Ton, the TolQ and TolR subunits, homologous to ExbB and ExbD, translocate protons through the IM and transfer force to TolA, homologous to TonB. At the OM, the PG binding lipoprotein Pal forms a complex with the soluble protein TolB that prevents Pal interaction with PG. Energized TolA disrupts the Pal-TolB complex and dissociates TolB from Pal, which is then free to interact with PG, stabilizing the association between the outer membrane and the underlying cell wall [5] (Figure 1).

The Ton and Tol-Pal systems can be hijacked by phages and bacteriocins that gain access to the periplasm through interaction with energized TonB or TolA and kill the infected cell with high efficiency [9,10]. Because of their high specificity, bacteriocins are attractive candidates for the development of new antimicrobial drugs [11–13].

The reported cryoelectron microscopy (cryo-EM) structures of the ExbBD and TolQR subcomplexes have a similar architecture with five ExbB/TolQ forming a pentameric ring that defines a hydrophobic central pore in which resides a dimer of the single transmembrane (TM) domain of ExbD/TolR (Figure 1) [14–17]. ExbBD and TolQR belong to a growing family of pmf-dependent molecular motors characterized by a 5 to 2 subunit stoichiometry [18]. Members of this family include MotAB that powers flagellar rotation, GldLM responsible for gliding motility, and ZorAB involved in the bacterial phage defense system [19–25]. The same 5:2 architecture is found for PomAB, the MotAB homolog that uses sodium ions instead of protons to drive flagellar rotation in Gram-positive bacteria [26,27]. ExbBD and MotAB derive from a common proton channel ancestor, and TolA is thought to have emerged from a TonB paralog during evolution [28,29].

MotAB is the most studied motor of the 5:2 family. Upon association of MotAB with the flagellum apparatus, the MotB periplasmic domain reaches and binds the PG layer, and the gated proton channel opens with the generation of a power stroke for each translocated proton [30,31]. Because of the rotational motion of the flagellum and structures of the MotAB complexes, it has been proposed that the MotA pentamer rotates around the MotB dimer tethered to the PG. In these mechanistic models, a highly conserved Asp residue on the MotB TM domain is responsible for proton translocation and the power stroke generated results in the rotation of the MotA pentamer by steps of 36° [18–20]. This Asp residue is also found on ExbD, TolR, and PomB and is absolutely required for activity. The rotation is then transmitted to the C-ring domain of the flagellum through transient interaction between the cytoplasmic domains of MotA and FliG in the C-ring [22]. Because of the extensive structural homologies between MotAB, ExbBD, and TolQR, the proton translocation mechanism and power stroke generation are likely similar, and the rotation model has been adapted for the Ton and Tol-Pal systems [32–34].

For Ton and Tol-Pal, it is hypothesized that energized TonB and TolA exert a pulling force perpendicular to the OM while bound to their protein partners. TonB binds to TonB-dependent transporter (TBDT) at the OM through a conserved N-terminal sequence on the TBDT called TonB box. The pulling exerted on TonB gradually unfolds the plug domain of the transporter, eventually opening a channel that allows the bound nutrient to enter the periplasm [35–37]. TolA interacts with the TolB-Pal complex. The pulling displaces TolB from Pal, and the retraction of the TolA periplasmic domain results in the deposition of TolB away from the OM and cell wall [5,38]. Ton and Tol-Pal-dependent bacteriocins are also hypothesized to gain entry into the cell via a pulling mechanism mediated by energized TonB or TolA [39,40].

**Figure 2:**
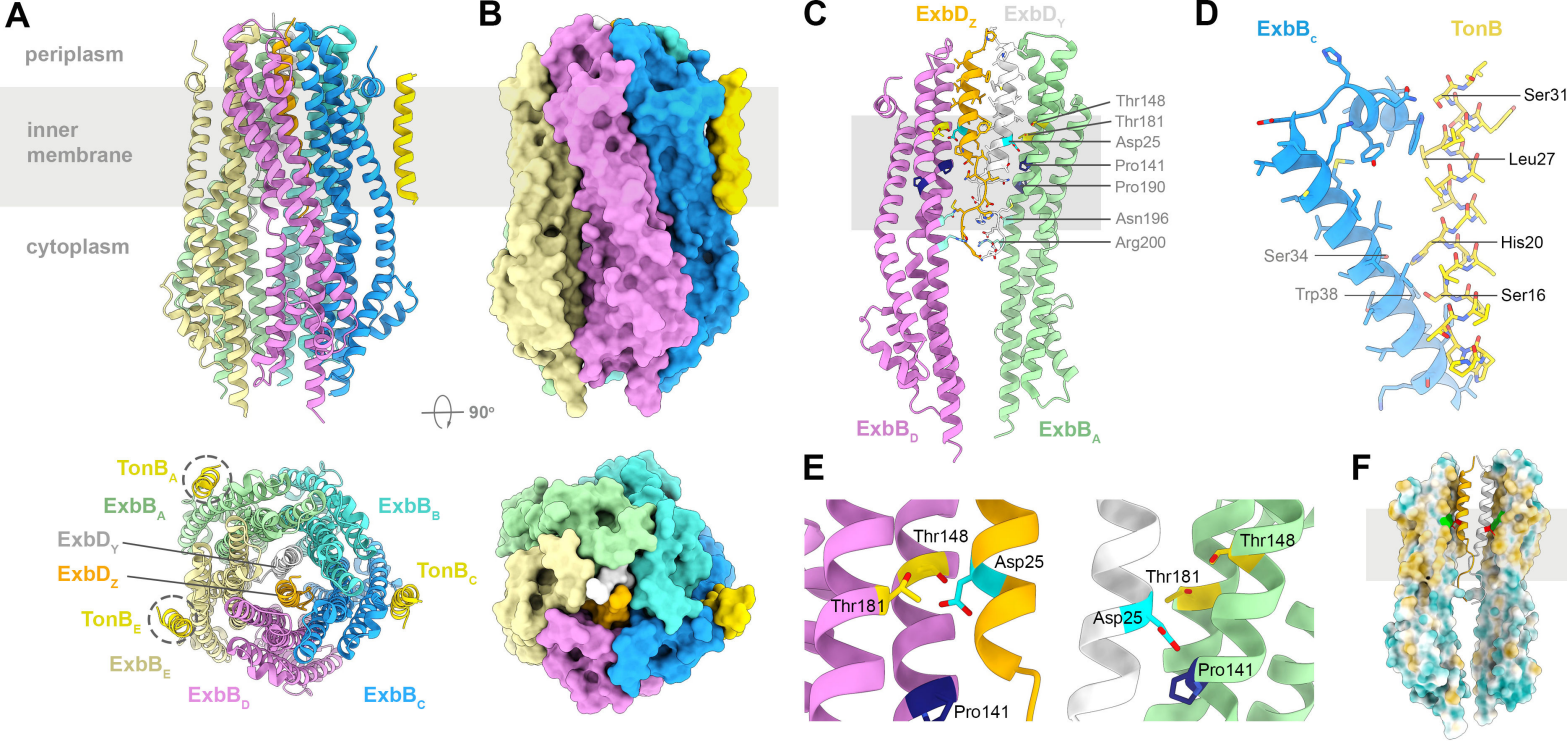
Structure of ExbBD-TonB. (**A**) Cartoon representation of ExbBD-TonB (PDB: 9DDO [33]). The inner membrane is represented by a grey rectangle. The alternate binding sites of TonB (from PDB 9DDP, 9DDQ [33]) are highlighted with dashed circles. (**B**) Molecular surface of ExbBD-TonB. (**C**) Cartoon representation of a cutaway view of ExbB_A_ and ExbB_D_ molecules with ExbD_Y_ and ExbD_Z_ in the hydrophobic pore, viewed from the membrane. ExbB helices α-5, α-6, and α-7 interact to form the hydrophobic pore and the hydrophilic cytoplasmic cavity. The essential Asp25, Thr148, and Thr181 are highlighted, together with other conserved residues. (**D**) Interacting residues in the ExbB-TonB complex. Only ExbB_C_ α-1 and TM of α-2 are shown for clarity. SHLS motif residues from TonB are labeled bold. (**E**) Close-up view of essential residues from panel C. (**F**) Cutaway view of surface colored from most hydrophilic (blue) to most hydrophobic (orange). The position of Asp25 is highlighted in red and Thr148, Thr181 in green. Images prepared with ChimeraX [42].

**Figure 3:**
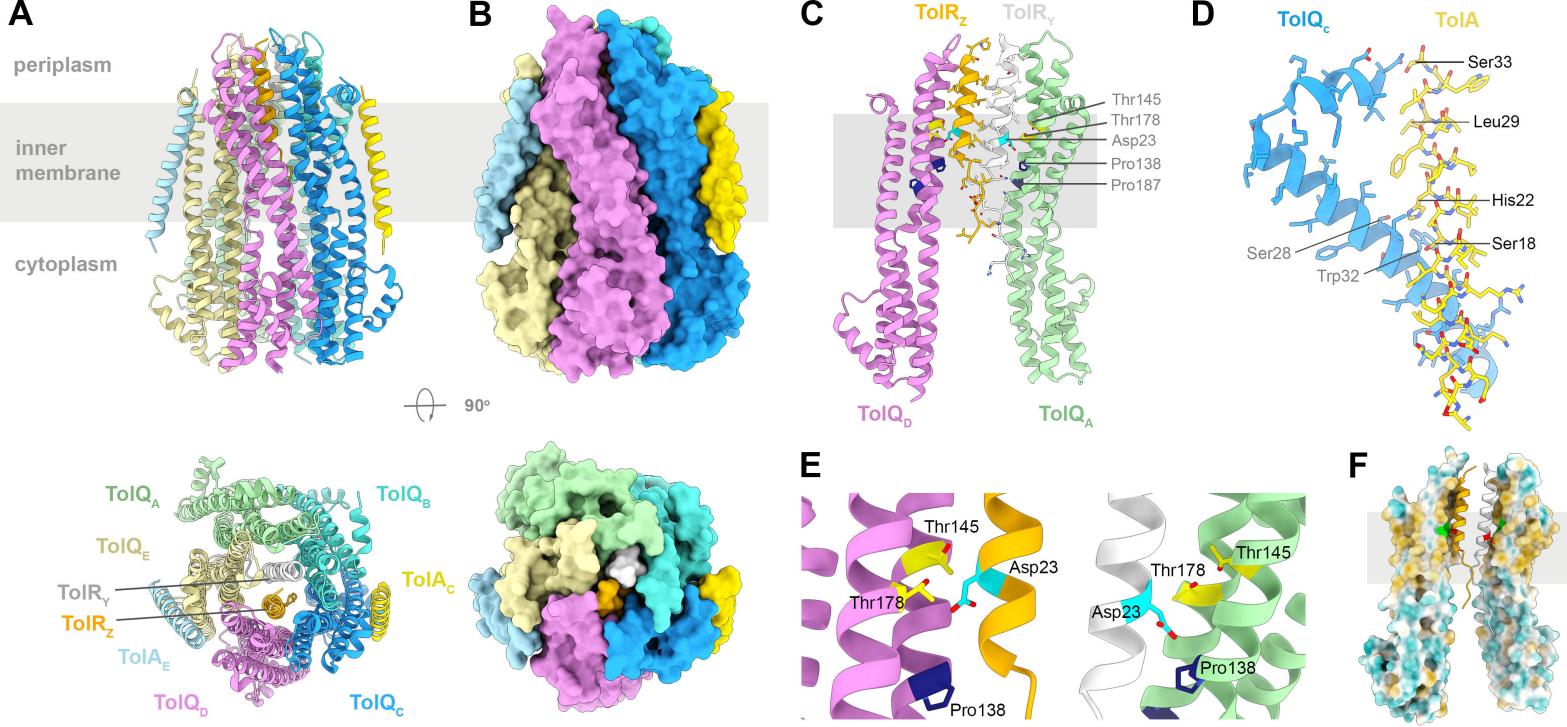
Structure of TolAQR. (**A**) Cartoon representation of TolAQR (PDB: 9DDM [33]). The inner membrane is represented by a grey rectangle. (**B**) Molecular surface of TolAQR. (**C**) Cartoon representation of a cutaway view of TolQ_A_ and TolQ_D_ molecules with TolR_Y_ and TolR_Z_ in the hydrophobic pore, viewed from the membrane. TolQ helices α-5, α-6 and α-7 interact to form the hydrophobic pore and the hydrophilic cytoplasmic cavity. The essential Asp23, Thr145 and Thr178 are highlighted, together with other conserved residues. (**D**) Interacting residues in the TolAQR complex. Only TolQ_C_ α-1 and TM of α-2 are shown for clarity. SHLS motif residues from TolA are labeled bold. (**E**) Close-up view of essential residues from panel C. (**F**) Cutaway view of surface colored from most hydrophilic (blue) to most hydrophobic (orange). The position of Asp23 is highlighted in red and Thr145, Thr178 in green.

Our structural knowledge of the Ton and Tol-Pal systems has greatly increased during the last decades [2,41]. Recent reports describe the structures of the *E. coli* TonB-ExbBD and TolAQR complexes, showing how TonB and TolA interact with the ExbBD and TolQR subcomplexes in the membrane (Figures 2 and 3) [32,33]. The periplasmic domains of TonB, TolA, ExbD, and TolR are not visible in these structures, suggesting that they are highly dynamic. The interaction between the TonB and ExbD periplasmic domains has also been investigated by X-ray crystallography and NMR: the ExbD periplasmic domains form a dynamic dimer, oscillating between a closed and open form, with the open form able to interact with TonB via the conserved D-box motif [43,44].

Despite all the structural and biochemical knowledge on Ton and Tol-Pal, it is still not understood precisely how these systems are activated, what modulates the gating of the proton channel, and how the force is produced and transduced between the two membranes. In this review, we discuss the latest structural information on the Ton and Tol-Pal motor complexes and how these data fit with the latest mechanistic models.

## Stoichiometry of TonB-ExbBD and TolAQR

The reported structures and stoichiometries of TonB-ExbBD, TolAQR motors (Figures 2 and 3) and their subcomplexes are summarized in Table 1 (low resolution negatively stained electron microscopy structures are not listed). Most of the ExbBD and TolQR subcomplexes share a 5:2 stoichiometry, except for *E. coli* ExbBD (*Ec*ExbBD) that range from 5:1, 5:2, and 6:3, while *Ec*ExbB alone can form hexamers [14,45,46]. The physiologically relevant stoichiometry of *Ec*ExbBD is likely 5:2 as it is observed for *S. marcescens Sm*ExbBD [15], homologous *A. baumannii Ab*TolQR [17], *Ec*TolQR [16], and the MotAB and PomAB cryo-EM structures [19,20,22,25,26]. The other stoichiometries observed for *Ec*ExbBD are likely due to experimental conditions and/or use of truncated subunits.

**Table 1:** Available structures and maps

Structure (*)	Stoichiometry	Organism	Res (Å)	pH	Method	PDB ID (**)
ExbB	5	*Escherichia coli*	2.6	7.0	X-Ray	5SV0 [45]
ExbBD	5:1	*Escherichia coli*	3.5	4.5	X-Ray	5SV1 [45]
ExbBD	5:2	*Escherichia coli*	3.3	7.5	Cryo-EM	6TYI [14]
ExbBD	5:1	*Escherichia coli*	7.1	5.4	Cryo-EM	5ZFV [46]
ExbBD	6:3	*Escherichia coli*	6.7	8.0	Cryo-EM	5ZFU [46]
ExbB	6	*Escherichia coli*	2.84	9.0	X-Ray	5ZFP [46]
ExbB	5	*Serratia marcescens*	3.2	8.0	Cryo-EM	6YE4 [15]
ExbBD	5:2	*Serratia marcescens*	4.0	8.0	Cryo-EM	7AJQ [15]
ExbBD	5:2	*Escherichia coli*	4.6	8.0	Cryo-EM	EMD-10902 [19]
ExbBD-TonB	5:2:1	*Pseudomonas savastanoi*	3.8	8.0	Cryo-EM	EMD-10897 [19]
ExbBD-TonB	5:2:1	*Escherichia coli*	2.8	7.5	Cryo-EM	9DDO [33]
ExbBD-TonB	5:2:1	*Escherichia coli*	3.16	7.5	Cryo-EM	9DDP [33]
ExbBD-TonB	5:2:1	*Escherichia coli*	3.19	7.5	Cryo-EM	9DDQ [33]
TolQ	5	*Acinetobacter baumannii*	3.02	8.0	Cryo-EM	9AVI [17]
TolQR	5:2	*Acinetobacter baumannii*	3.34	8.0	Cryo-EM	8VLW [17]
TolQR	5:2	*Escherichia coli*	4.2	8.0	Cryo-EM	8ODT [16]
TolQR	5:2	*Escherichia coli*	2.92	7.8	Cryo-EM	9O40 [47]
TolQR A_TEV_	5:2:2	*Escherichia coli*	2.94	7.5	Cryo-EM	9DDM [33]
TolQR A_TEV_	5:2:2	*Escherichia coli*	3.18	7.5	Cryo-EM	9DDN [33]
TolQR A	5:2:2	*Escherichia coli*	5.2	7.5	Cryo-EM	EMD-70142 [33]
TolQR A	5:2:1	*Escherichia coli*	3.6	7.5	Cryo-EM	9K49 [32]
TolQR A	5:2:1	*Escherichia coli*	4.19	7.5	Cryo-EM	9KCH [32]
TolQR A	5:2:3	*Escherichia coli*	3.52	7.8	Cryo-EM	9QVD [47]
TolQR A	5:2:1	*Escherichia coli*	3.28	7.8	Cryo-EM	9QUQ [47]
						
ExbD (43-141)	1	*Escherichia coli*		3.0	NMR	2PFU [48]
ExbD (43-140)	2	*Serratia marcescens*		7.0	NMR	8PEK [44]
ExbD-TonB (61–141:40–62)	2:1	*Escherichia coli*	1.52	7.0	X-Ray	8P9R [44]
ExbD-TonB (59–141:43–52)	2:1	*Escherichia coli*	1.42	4.6	X-Ray	8VGC [43]
ExbD-TonB (59–141:43–51)	2:1	*Escherichia coli*	1.42	5.8	X-Ray	8VGD [43]
TolR (62-133)	2	*Haemophilus influenzae*		6.7	NMR	2JWK [49]
TolR (62-133)	2	*Haemophilus influenzae*		6.7	NMR	2JWL [49]
TolR (36-142)	2	*Escherichia coli*	1.70	7.5	X-Ray	5BY4 [50]

(*) numbers in parentheses correspond to sequence numbering of truncated proteins(**) Electron microscopy database (EMD) ID provided in case no atomic model was deposited in the PDB.

ExbBD and TolQR have a central hydrophobic pore where proton translocation presumably takes place. The pore is lined by the ExbB and TolQ TM domains 2 and 3, surrounding a dimer of the TM domains of ExbD and TolR tucked into the pore. Because of the mismatch between the five and two subunits, the pentamers are not symmetrical. The hydrophobic pores are not in register with the IM but shifted 1 nm toward the periplasmic space. In most of the structures, the essential Asp on the dimer of ExbD and TolR TM domains points in opposite directions, with one Asp engaged in interaction with a conserved Thr on ExbB and TolQ, while the second Asp points toward the cytoplasmic hydrophilic cavity defined by the pentamer (Figures 2C–E , and 3C–E). This asymmetric arrangement is also found for the MotAB and PomAB complexes and is likely important for the directionality of the proton flux and generation of the power stroke [18,51]. Intriguingly, a different arrangement is observed in the *Ab*TolQR structure in which both Asp are close to the cytoplasmic cavity and away from the conserved Thr on TolQ [17]. It is not clear why this arrangement is different in *Ab*TolQR, but it could represent a structural intermediate in the proton translocation cycle.

ExbD and TolR both have one TM domain followed by an internally disordered periplasmic linker of about 20 residues and a folded C-terminal domain (Supplementary Figure S1B). The periplasmic domains of ExbD and TolR are not visible in the ExbBD and TolQR structures, suggesting they are highly flexible. Only in the cryo-EM map of *Ab*TolQR is the TolR periplasmic region partially visible at low contour levels as a low-resolution globular density that could fit a dimer of TolR C-terminal domains [17]. The distance between the globular density and the IM plane is about 50 Å, suggesting that the TolR periplasmic linker is extended.

**Figure 4:**
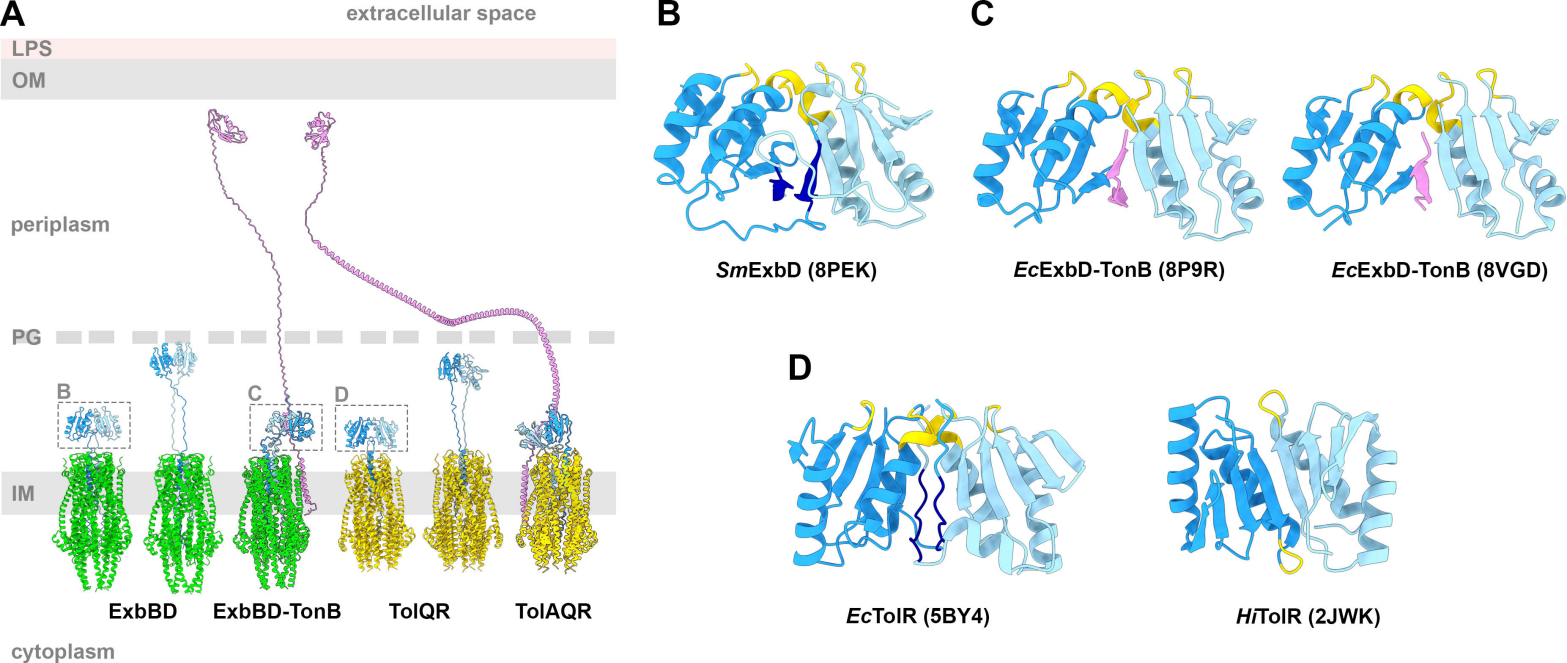
Structures of the Ton and Tol systems in different states. **(A**) Alphafold3 composite models of three states of each of the ExbBD and TolQR complexes: closed, PG-binding and binding TonB/TolA, respectively [61]. The ExbB pentamers are shown in green, TolQ pentamers in gold, ExbD and TolR dimers in shades of blue, while TonB and TolA are shown in pink. The PG-binding models are derived from the AF3 models. (**B**) Structure of the periplasmic *Sm*ExbD domain [44]. Monomers are shown in shades of blue, with the peptidoglycan-binding region highlighted in yellow and NIBS in dark blue. (**C**) Structures of the periplasmic *Ec*ExbD domain bound to the D-box of TonB (in pink) [43,44]. (**D**) Structures of the periplasmic domains of TolR [49,50].

The TonB and TolA subunits are pivotal components of the Ton and Tol-Pal systems as they convey the energy of the pmf from the IM to the OM. Like ExbD and TolR, TonB and TolA have a single TM helix, followed by a periplasmic linker and a folded C-terminal domain (Supplementary Figure S1C). The sequences of the TonB and TolA linkers are significantly longer than for ExbD and TolR (~115 residues for *Ec*TonB, 300 for *Ec*TolA), which should allow the C-terminal domains to reach the OM as illustrated in Figures 1 and 4A. The *Ec*TonB linker contains segments with multiple prolines that might organize as a polyproline helix II, conferring some rigidity to the linker [52]. The *Ec*TolA linker contains multiple stretches of poly-alanine that are predicted to form elongated coiled-coil α-helices (Supplementary Figure S1C). It is noteworthy that the poly-alanine repeats of *Ec*TolA are not a conserved feature, as in other species, the TolA linker has instead proline-rich segments like the one found in *Ec*TonB [28].

TonB and TolA bind different classes of proteins at the OM through their folded C-terminal domains. While *Ec*TonB and *Ec*TolA share less than 20% identity, their C-terminal domains are structural homologs (Supplementary Figure S1C) [53]. Furthermore, they both interact with their OM partners by ß-strand augmentation. In both cases, the disordered TBDT TonB box and N-terminal region of TolB fold into a parallel ß-strand upon interaction with the C-terminal ß-strand of TonB and TolA [38,54–56].

The TM domains of TonB and TolA share extensive homologies, and both contain the conserved Ser-3X-His-6X-Leu-3X-Ser (SHLS) motif. The cryo-EM structures of *E. coli* TonB-ExbBD and TolAQR show how the TonB and TolA TM domains in the membrane interact at the periphery of ExbBD and TolQR (Figures 2 and 3) [32,33]. The mode of interaction is similar, with the TonB/TolA TM contacting both the TM1 and the small periplasmic N-terminal amphipathic helix of ExbB/TolQ. Ser and His of the **SH**LS motif form a hub of interactions with TM1 of ExbB and TolQ while the last Ser (SHL**S**) interacts with the N-terminal amphipathic helix (Figures 2D and 3D). TolA Leu (SH**L**S) interacts either with TM1 of TolQ and/or TM1 of a neighboring TolQ subunit, while on TonB Leu is not engaged in any interaction with ExbB [33]. In both TonB-ExbBD and TolAQR, a network of hydrophobic interactions connects the Ser and His **SH**LS motif with conserved Ser and Trp on ExbB or TolQ TM1, and a conserved Pro on TM3. This network of conserved residues is suspected to be responsible for the signaling of TonB and TolA engaged with OM components [33]. Conformational changes at the interface between TonB and ExbB or TolA and TolQ would be propagated to the conserved Pro on TM3, eventually opening the proton channel [33].

The stoichiometry of the *Ec*TonB-ExbBD complex is 1:5:2. Three structures have been reported, in which the TonB TM binds three distinct ExbB subunits on the pentamer (PDB 9DDO, 9DDP, and 9DDQ) (Figure 2A–B) [33]. Comparison of TonB-ExbBD with ExbBD (PDB 6TYI [14]) does not reveal noticeable conformational changes for ExbBD, suggesting that the binding of TonB alone is not sufficient to open the proton channel.

Two different stoichiometries have been reported for *Ec*TolAQR, either 2:5:2 or 1:5:2 [32,33](Figure 3). For the 1:5:2 stoichiometry, the authors report a preliminary 3D map with a 2:5:2 ratio, which was further processed by 3D classification and resulted in two 1:5:2 structures at 3.6 Å and 4.2 Å resolution, respectively (PDB 9K49, 9KCH [32]). These two structures differ in TolA interacting with two distinct TolQ on the pentamer and were interpreted as two conformational states of the TolAQR complex during the transfer of energy [32]. However, careful inspection of the 3.6 Å and 4.2 Å resolution cryo-EM 3D maps (EMD-62050 and EMD-62251) reveals weak residual densities at the two distinct TolA binding sites, which could indicate a mixture of 1:5:2 and 2:5:2 complexes.

Except for the additional TolA subunits, the 1:5:2 3.6 Å (PDB 9K49 [32]) and 2:5:2 2.9 Å (PDB 9DDM [33]) resolution structures are remarkably similar with an RMSD of 0.767 Å for 6882 common atoms (Figure 3A–B).

Two structures were reported for the 2:5:2 TolAQR complex (9DDM and 9DDN at 2.9 Å and 3.2 Å resolution), highlighting dynamic conformational changes of the cytoplasmic domains of TolQ (see suppl movie in [33]). The two 2:5:2 TolAQR 3D cryo-EM maps show well-defined densities for the two TolA TM domains, arguing for a major 2:5:2 population. Furthermore, weak densities for an additional TolA TM domain are visible in the unsharpened cryo-EM 3D map, suggesting partial occupation at a third binding site on the pentamer (see suppl Figure 10-D in [33]). For both Ton and Tol-Pal systems, a functional TonB-ExbBD or TolAQR complex must contain at least one TonB or TolA subunit. Because of their extensive homologies and cross-complementation, it is intriguing that the two complexes could differ in their respective stoichiometries. The buried surface area at the TolA-TolQ interface is more extended than for TonB-ExbB (780 Å^2^ vs 480 Å^2^), indicative of a more stable interaction between TolA and TolQR. The reported estimated copy numbers per cell in physiological conditions are 650 TonB, 2000 ExbB, and 950 ExbD for TonB-ExbD, and 500 TolA, 1700 TolQ, and 900 TolR for TolAQR [57]. While these numbers do not reflect perfectly the 5:2 ratio observed for the ExbBD and TolQR structures, the fact that ExbB/TolQ >ExbD/TolR >TonB/TolA agrees well with a 1:5:2 ratio for the full TonB-ExbBD and TolAQR complexes. The overexpression of *tolA* might result in an excess of TolA compared with TolQR, leading to an artifactual TolA binding at more than one site on TolQR. Nevertheless, the 2:5:2 TolAQR complex could be physiologically relevant for some Tol functions. Tol-Pal is involved in multiple processes comprising maintenance of cell envelope integrity, lipid homeostasis, PG remodeling, and cell division [5]. During cell division, TolAQR accumulates at cell constriction sites. TolA and TolQ are recruited independently to the divisome through interaction with different proteins of the cell division machinery [58–60]. This could lead to a higher concentration of TolA, favoring the formation of 2:5:2 complexes.

Interestingly, the TonB and TolA binding sites on ExbBD and TolQR, including the partially occupied TolA third binding site, are found at equivalent positions on the ExbB and TolQ pentamer (Figures 2A and 3A). This observed preferential binding suggests a similar mechanism of assembly for TonB and TolA with their respective ExbBD and TolQR subcomplexes. A possible explanation has been proposed in which the interaction between TonB/TolA and ExbD/TolR in the periplasm dictates the preferential binding of TonB/TolA with ExbB/TolQ in the IM (see Figure 4 in [33]).

## Role of the ExbD and TolR periplasmic domains

As mentioned earlier, ExbD and TolR have an unstructured periplasmic linker about 20 residues in length followed by a folded C-terminal domain that contains a conserved PG binding site (Supplementary Figure S1B).

NMR studies of the *Sm*ExbD soluble periplasmic domain show that it forms a dynamic dimer in solution, oscillating between a closed and open form [44]. In the closed form, a conserved motif of the linker region, the N-terminal Intermolecular Beta Strand (NIBS), assembles as a swapped anti-parallel ß-sheet (Figure 4B). The NIBS motif has been found essential for Ton activities [62]. In the open form, the NIBS is no longer structured, and the linker is disordered. Topologically, the closed form would spatially constrain the ExbD periplasmic dimer close to the TM domains, potentially occluding the proton channel, while in the open form, the folded dimer can extend toward the periplasm, eventually able to reach the PG layer (Figure 4A). The open form of the dimer is also able to interact with a conserved motif on TonB called the D-box, which in *E. coli* is located about 15 residues upstream of the TM domain (Supplementary Figure S1C) [43,44]. Upon binding, the unstructured D-box motif forms a parallel ß-sheet with the C-terminal ß-sheet of one ExbD protomer and an anti-parallel ß-sheet with the C-terminal ß-sheet of the other protomer (Figure 4C). In the closed form, the D-box binding site is masked by the swapped NIBS.

The X-ray structure of the *Ec*TolR soluble periplasmic domain shows a similar closed dimer organization, including the swapped NIBS in the linker region (Figure 4D) [50]. Because TolA also has a conserved R-box motif equivalent to the TonB D-box, it is speculated that TolA interacts with the equivalent open form of the TolR dimer [43,44].

A different dimeric organization has been reported for *H. influenzae Hi*TolR (Figure 4D) [49]. However, in this latter study, both the NIBS and C-terminal ß-sheet of the folded domain of *Hi*TolR have been removed, casting doubt on the relevance of the observed dimeric interface for this construct.

Because the NIBS and TM regions of ExbD and TolR are separated by only five residues, the periplasmic dimer must be sitting on top of the membrane-embedded ExbBD and TolQR subcomplex (Figure 4A). However, none of the reported 3D cryo-EM maps of TonB-ExBD, TolAQR, ExbBD, and TolQR exhibit densities for the closed form of the dimer, suggesting that the dimer is highly flexible and most likely in its open conformation. It remains to be seen if the closed form, with the swapped NIBS, exists in the context of the full ExbBD and TolQR complexes.

Like Pal and MotB, ExbD and TolR are OmpA-like proteins, a family of proteins involved in the binding of the PG cell wall. The soluble *Sm*ExbD periplasmic construct binds to PG sacculi, but the interaction disrupts the dimeric interface [44]. The soluble *Ec*TolR dimer does not show PG binding activity, but a truncated version of *Ec*TolR missing the linker and part of the C-terminal folded region was found to interact with PG [50]. Therefore, for both ExbD and TolR, the dimeric interface needs to be disrupted for the binding to PG to occur.

In the periplasm, the distance between the IM and PG cell wall is about 90 Å [1]. For the ExbD or TolR to reach and interact with the PG cell wall, the periplasmic linker must be fully extended (Figure 4A). About 10 to 15 residues separate the TM helix of TonB or TolA from their respective D-box or R-box motif. Due to topological constraints, neither the closed form of the dimer nor the open form in complex with the TonB D-box or TolA R-box would be able to reach the PG layer (Figure 4A).

## Mechanistic models for Ton and Tol-Pal

Ton and Tol-Pal are sophisticated molecular motor systems that are challenging to study. The TonB-ExbBD and TolAQR have now been purified, allowing their structures to be determined, representing a significant step to understand how these molecular engines work [32,33]. However, there is so far no *in vitro* activity test that would allow validation of a precise mechanism as has been done for the F1-ATPase rotation mechanism [63]. For Ton and Tol-Pal, as well as the Mot system, the gating of the proton channel, power stroke generation, and transfer of energy are dependent on an intact cell envelope comprising the IM, OM, and PG layers. Therefore, the effects of mutation or chemical modification on specific activity can only be tested *in vivo*.

Based on the structure of the vitamin B12 transporter BtuB bound to a C-terminal fragment of TonB, a *pulling* model was introduced in which energized TonB exerts a pulling force on the TBDT TonB box, perpendicular to the OM plane and toward the periplasm [56]. The pulling would gradually unfold the plug domain of the TBDT, eventually opening a channel through which the bound nutrient diffuses into the periplasm. The *pulling* model was validated *in silico* by molecular dynamics, and *in vitro* through single molecule force spectroscopy measurements using an immobilized TBDT and TonB attached to an AFM probe [36,37]. A similar pulling mechanism is likely to take place in the Tol-Pal system, in which the folded TolA C-terminal domain binds the TolB-Pal complex and dissociates TolB from Pal by pulling TolB away from Pal [38]. Ton and Tol-Pal-dependent colicins enter the cell by unfolding and threading through specific OM proteins [39,40,64,65]. The translocation process driven by TonB or TolA bound to the TonB or TolA box of the colicins most likely uses a similar pulling mechanism.

In the Mot system, the rotation of the MotA pentamer around MotB is the most plausible mechanism for rotation of the flagellum [19,20]. Because of the structural homologies between MotAB, ExbBD, and TolQR in the pore region where consumed energy generates a power stroke, the rotation model is most likely used by Ton and Tol-Pal.

**Figure 5:**
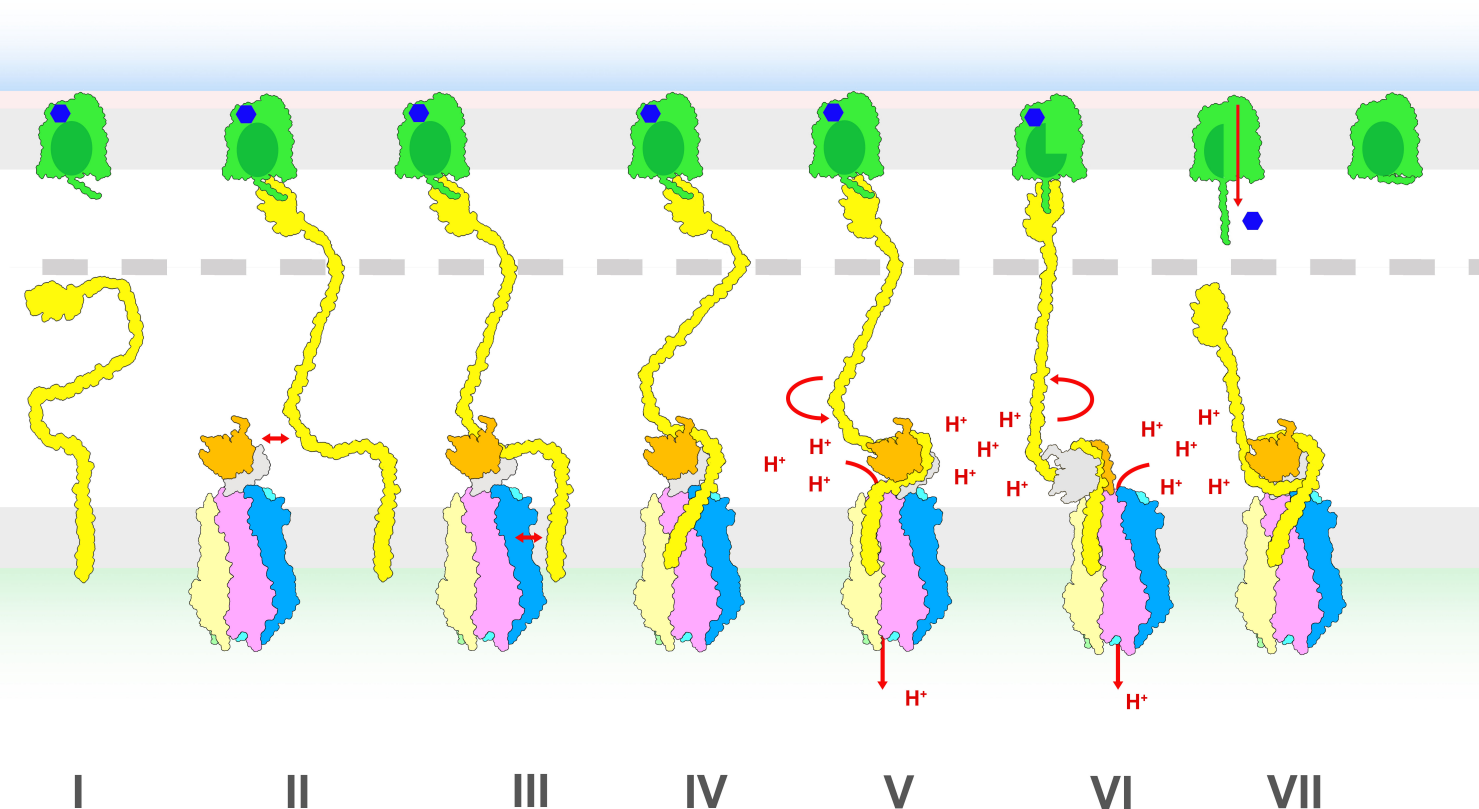
Ton system hypothetical *pull and wrap* mechanistic model. (**I**) TBDT (green) in the OM (grey rectangle) binds a nutrient molecule (blue hexagon) inducing the TonB box to extend into the periplasm, while TonB (yellow) freely diffuses in the IM. The outer membrane is lined by LPS (pink rectangle) towards the extracellular space (blue gradient), the periplasm harbors a PG layer (grey dashed line) and the cytoplasm is represented by a green gradient. (**II**) The TBDT TonB box binds the C-terminal domain of TonB, while the TonB D-box region approaches the ExbD periplasmic dimer (orange and grey) from the ExbBD subcomplex in the IM. (**III**) The ExbD periplasmic dimer binds the TonB D-box. (**IV**) The IM ExbB pentamer binds the TonB TM forming a full TonB-ExbBD-TBDT complex. (**V**) Diffusion of the complex in the membrane pulls the periplasmic linker of TonB causing a reorientation of the TonB TM on ExbBD, a conformational change in ExbB TM3 that opens the pentameric pore in ExbBD. The pmf drives proton translocation through the pore and rotation of the ExbD TM dimer. (**VI**) By this rotation the TonB periplasmic linker wraps around the ExbD dimer, pulling the C-terminal domain of TonB toward the IM. (**VII**) Due to the pulling force exerted on the TonB box, the plug domain of TBDT partially unfolds and opens a channel through the TBDT barrel permitting the bound nutrient to diffuse into the periplasm (red arrow). Further unfolding of the plug domain generates resistance to the pulling force and the C-terminal domain of TonB detaches from the TonB box, releasing the tension of the linker. With the proton channel closed the TonB-ExbBD complex resets to the ground state. The TBDT plug domain refolds and retracts into the barrel.

How is the rotation of ExbB/TolQ around ExbD/TolR in the IM transmitted to TonB/TolA to exert a pulling force on proteins at the OM? In the *wrap and pull* model, the binding of TonB to a nutrient-loaded TBDT at the OM signals the opening of the proton channel (Figure 5) [33,34]. TonB alone (Figure 5-I) first binds to a nutrient-loaded TBDT (Figure 5-II). The D-box on TonB then interacts with the ExbD dimer in the periplasm (Figure 5-III) and the TonB TM domain binds to the nearest ExbB subunit in the membrane (Figure 5-IV). This ordered sequence of events for the assembly of the TBDT-TonB-ExbBD full complex would explain the observed preferential interaction of TonB at three different positions on the ExbB pentamer (Figure 2A) [33]. The opening of the proton channel would be triggered by a change of orientation of the TM of TonB that leads to the widening of the pentameric pore (Figure 5-V). The rotation of ExbD compared with ExbB and TonB results in the wrapping of the TonB linker around ExbD, exerting a pulling force on the TonB C-terminal domain bound to the TBDT TonB box (Figure 5-VI). As the TonB box is pulled into the periplasm, the plug domain gradually unfolds, creating a channel within the TBDT through which the nutrient diffuses and enters the periplasm. At some point, the force required to further unfold the plug domain becomes greater than the pulling force exerted. The TonB C-terminal domain then detaches from the TBDT TonB box and the plug domain folds back into its original state (Figure 5-VII) [66].

Following their study on the *Sm* and *Ec*ExbD periplasmic fragments, Zinke et al. have proposed a different version of the *wrap and pull* model in which the closed form of the ExbD dimer maintains the proton channel in a closed configuration [44]. The transition of the dimer into its extended open form allows ExbD to reach and interact with the PG layer, leading to the dissociation of the dimer. TonB inserts in between the two ExbD, and the dimer reassociates in its open form while interacting with the D-box of TonB. The rotation of ExbD relative to TonB and ExbB then follows the *wrap and pull* model [44]. In this model, the gating of the proton channel depends on the equilibrium between the closed and open forms of the ExbD dimer, not on the interaction of TonB with a TBDT. It is not clear how the complex reverts to its resting state, as TonB is locked in between the two ExbD, preventing the ExbD dimer to fold back into the closed conformation.

In the Mot system, MotB binds to the PG and the pentamer of MotA rotates around the immobilized MotB. By analogy, ExbD and TolR have been thought to also interact with the PG layer and act as stators. In the different versions of the *wrap and pull* models described, ExbD does not bind to the PG during the energy consumption cycle, and it is TonB that is tethered to the OM and acts as a stator. While it has been shown that ExbD and TolR can bind PG *in vitro*, it is not clear if the interaction with PG is required for Ton or Tol-Pal activity [44,50].

Based on the *Ab*TolQR structure, Karimullina et al. proposed a mechanistic model that is equivalent to the *wrap and pull* model, with the TolA periplasmic linker threading in between the two TolR periplasmic domains [17]. However, in the schematic representation supporting the mechanism (see figure 9 in [17]), TolR is bound to the PG during the whole cycle, even while TolA interacts with the TolR dimer through its R-box, which is topologically unlikely (Figure 4) [17].

A different mechanistic model, the *turn and reset* model, was introduced by Yeow et al. [32]. In this model, the two observed structures of the *Ec*TolAQR complex, differing by the position of TolA on the TolQ pentamer, correspond to distinct conformational states. In state A, TolA binds one TolQ in the membrane and TolR in the periplasm through its R-box domain. The first proton translocation event leads to a 36° clockwise rotation of the pentamer, with the two Asp on the TolR TM exchanging their protonated states, resulting in state B. The second proton translocation induces a second clockwise rotation of the pentamer, but while TolA is still interacting with TolR in the periplasm, the TM of TolA detaches from TolQ and is repositioned on the next TolQ in the membrane, reverting to state A (see Figure 4 in [32]). In the periplasm, the linker of TolA is in a compact state. The A to B transition results in a reorganization of the TolA linker into an extended conformation allowing TolA to reach and interact with the TolB-Pal complex. Upon reversion to state A, TolA folds back into its compact conformation, shortening the linker and displacing TolB from Pal. The periplasmic TolA linker plays a central role in the *turn and reset* model, and replacement of this linker with an unrelated polypeptide would most likely lead to the loss of pmf-dependent Tol-Pal activities. However, *in vivo* experiments show that such activities are still detected with chimeras of TolA: replacement with the TonB linker still supports cellular growth in the presence of SDS, albeit with less efficiency, and cells remain sensitive to Tol-Pal pmf-dependent colicins using an unstructured linker [16]. Again, in the schematic supporting the mechanistic model (see Figure 4C in [32]), the TolR dimer interacts both with the PG and the TolA R-box, which is unlikely as the interaction with the R-box restrains the TolR dimer from reaching the PG layer (Figure 4A).

Because of the cross-complementation between Ton and Tol-Pal, these two systems are likely to share a similar mechanism of action. In this respect, the *turn and reset* model might not be adopted by the Ton system since the TonB linker is not predicted to have a compact form. The *wrap and pull* model remains largely speculative as well, and it is not clear how the linker of TonB wraps around ExbD during the rotation. The NIBS, D-box and R-box, and ExbD and TolR binding to the PG need further scrutiny to understand their respective contributions. Mutation experiments have shown the importance of NIBS for Ton activities [62]. It is not yet known if the TonB D-box, TolA R-box, and ExbD and TolR PG binding domains are required for Ton and Tol-Pal activities. Finally, the gating of the proton channel is not yet understood. The design of an *in vitro* experiment to test for the activity of TonB-ExbBD and TolAQR, or the ExbBD and TolQR subcomplexes, would be a landmark for understanding how these proteins work.

## Summary

In recent years, there have been notable advances in the understanding of the architecture and mechanism of the Ton and Tol-Pal molecular machines. These studies revealed additional details of the high-resolution structures of the components of these systems, as well as details of their possible mechanism. With such a dynamic complex that spans the periplasm, linking components of the IM to the OM, it is difficult to capture all interacting components at once. Cryo-EM tomography on whole bacterial cells could help to understand the architecture of the intact systems. Determining the high-resolution structures of Ton or Tol-Pal mutants might also give valuable insights.

**Perspectives**

- Understanding how the Ton and Tol-Pal molecular motors convert energy derived from the PMF at the inner membrane into force at the outer membrane can provide the framework for inhibitor design against this essential machinery conserved across Gram-negative bacteria.
- In the current Ton models, the binding of a nutrient to a TonB-dependent transporter (TBDT) leads to the unmasking of the TonB box. The disordered TonB box then interacts with TonB, signaling the nutrient-loaded status to the ExbBD subcomplex at the IM. However, some TBDTs have been found to interact with TonB in the absence of nutrient, suggesting that TonB could be involved in some regulatory process. In this respect, it would be interesting to determine the structures of TBDT-TonB complexes in the presence and absence of nutrient.
- It is still poorly understood how these systems are activated. Are the interactions between TonB or TolA with their respective TBDT or TolB-Pal at the OM triggering a signal that opens the proton channel at the IM? Or is the pmf used to allow TonB and TolA to reach their targets? Future research will focus on a better understanding of the interactions between the periplasmic components of the machinery and perhaps capturing the whole extended complex in the cell.

### Note

During the revision process of this mini review, additional structures of the *E. coli* TolAQR complexes have been reported by Shen et al. [47]. In these structures, either one or three copies of TolA were found per complex, suggesting that TolQR can associate with multiple TolA protomers.

## Supplementary material

online supplementary material 1

## Open Access

Open access for this article was enabled by the participation of National Institutes of Health (NIH) in an all-inclusive Read & Publish agreement with Portland Press and the Biochemical Society.

## Abbreviations

IM: inner membrane
OM: outer membrane
PG: peptidoglycan
TM: transmembrane
cryo-EM: cryoelectron microscopy
pmf: proton motive force
