# Supplementary material for: Advances in understanding Ton and Tol system motor proteins
Source: Biochem J. 2026 Jan 28;54(Pt 1):107–19. doi: 10.1042/BST20253128 (PMC12905483; doi:10.1042/BST20253128)
Supplement: online supplementary material 1 [file bcj-54-1-BST20253128-s001.docx]

Supplementary information for

**Advances in understanding Ton and Tol system motor proteins**

Herve CELIA, Susan K. BUCHANAN, Istvan BOTOS

Laboratory of Molecular Biology, National Institute of Diabetes and Digestive and Kidney Diseases, National Institutes of Health, Bethesda, MD 20892, USA.

**Supplementary figure: Pairwise alignments and conservation scores mapped on structure predictions of *Ec*ExbB and *Ec*TolQ (A), *Ec*ExbD and *Ec*TolR (B) and *Ec*TonB and *Ec*TolA (C).**

Pairwise alignments performed on the EMBL-EBI website (https://www.ebi.ac.uk/jdispatcher/psa)[1]: “|” represent identical residues, “:” similar residues, “.” are variable.

Conservation scores were determined with ConSurf (https://consurf.tau.ac.il/consurf_index.php)[2], with highly conserved residues in maroon, average in white, and poorly conserved in turquoise. The underrepresented residues are in yellow.

Secondary structures are represented as follow:
α-helix
ß-sheet

TM region

The alphafold prediction 3D structures are shown as cylinder and stub cartoons. The residues are color coded according to their conservation score determined with ConSurf. The figures were prepared with ChimeraX[3].

(**A**) **ExbD-TolQ**: the TM1, TM2 and TM3 of ExbB and TolR are indicated.

(**B**) **ExbD-TolR**: the essential Asp on ExbD and TolR TM domains are highlighted in red on the pair aligment panel, and the NIBS motifs are shown. These regions are also shown on the cartoon representations.

(**C**) **TonB-TolA**: the conserved SHLS motif on TonB and TolA TM are highlighted in red on the pair aligment panel, and the D-box and R-box motifs are shown. These regions are also shown on the cartoon representations.

**A**

**
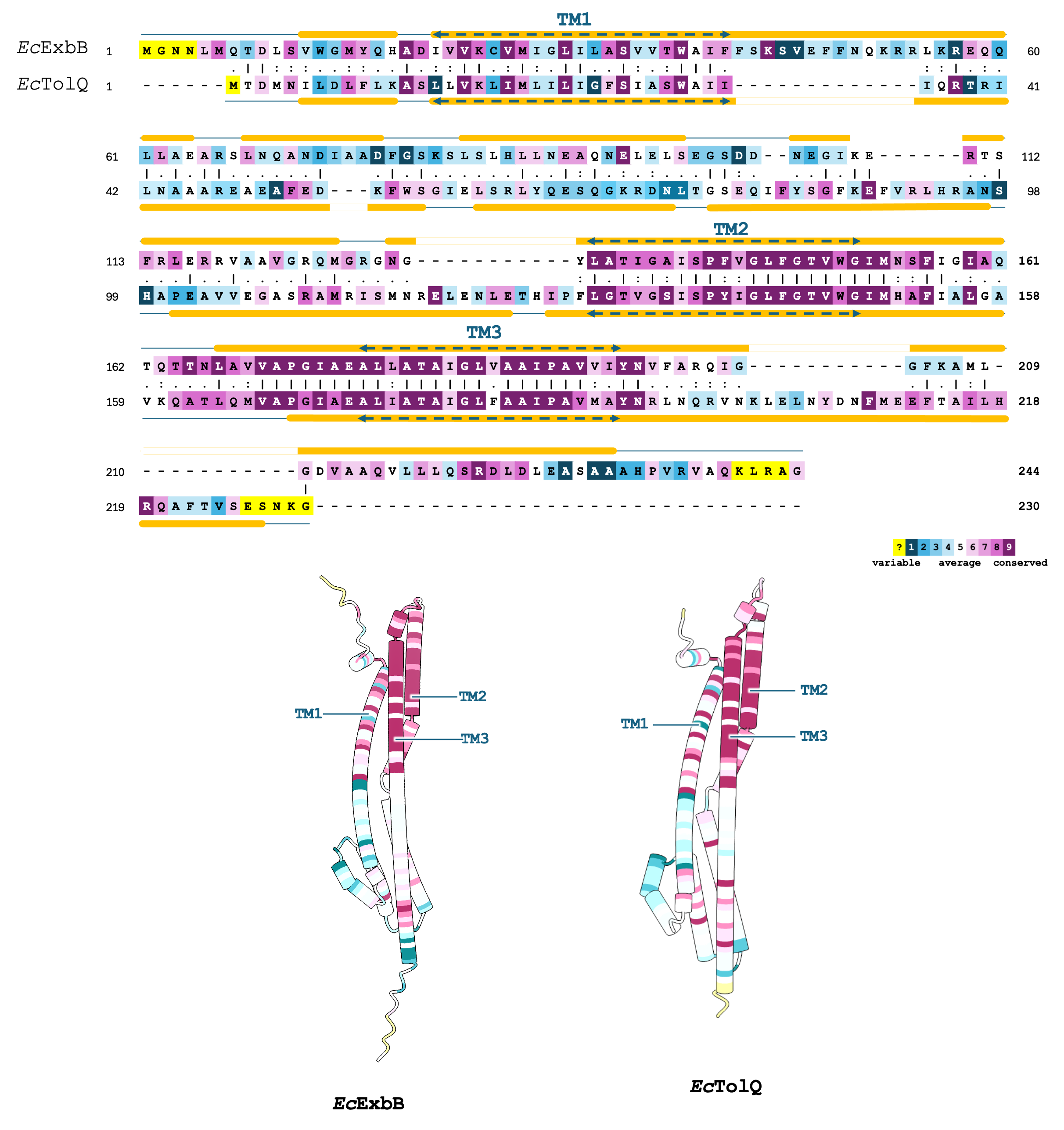
**

**B**

**
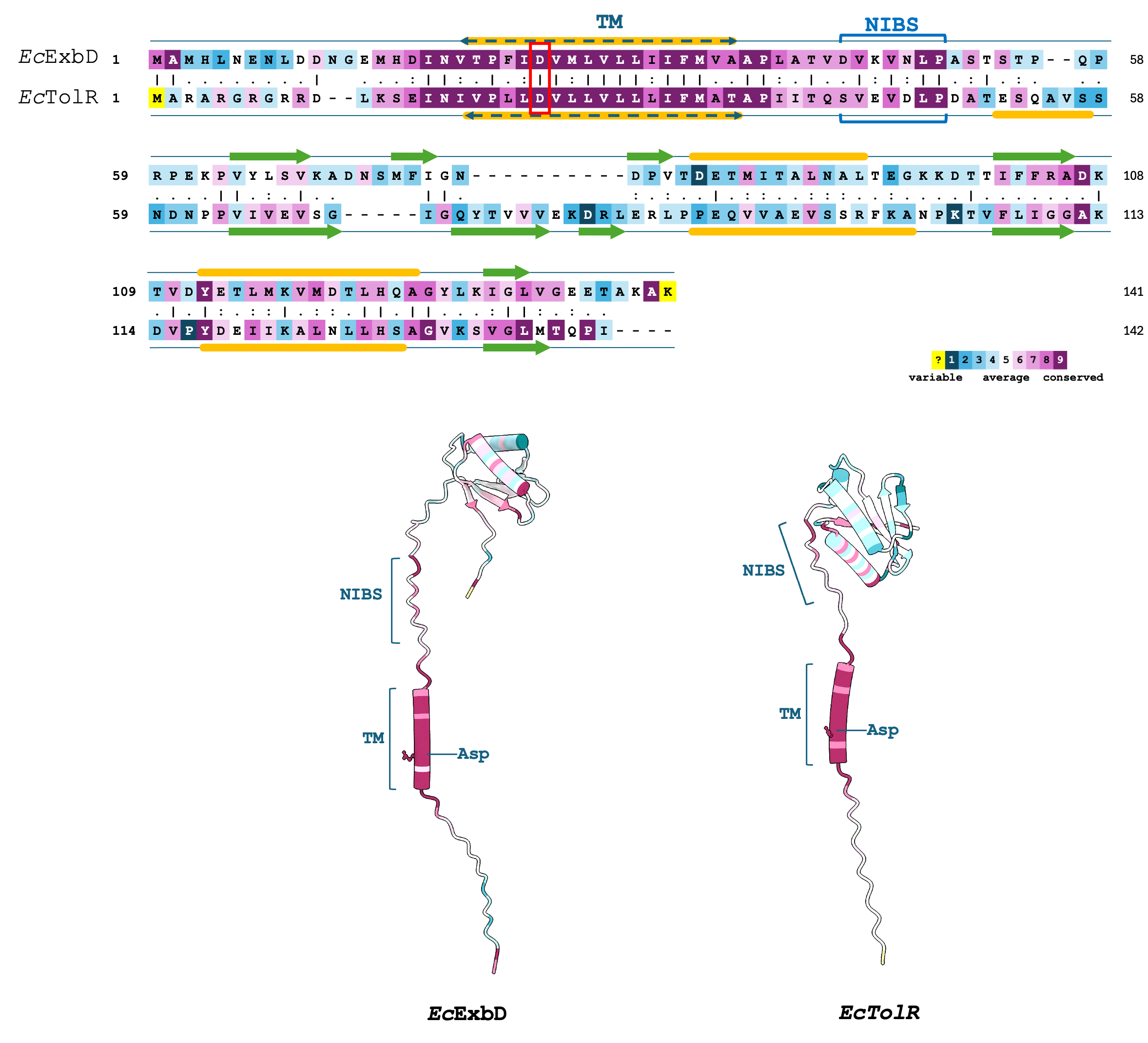
**

**C**

**
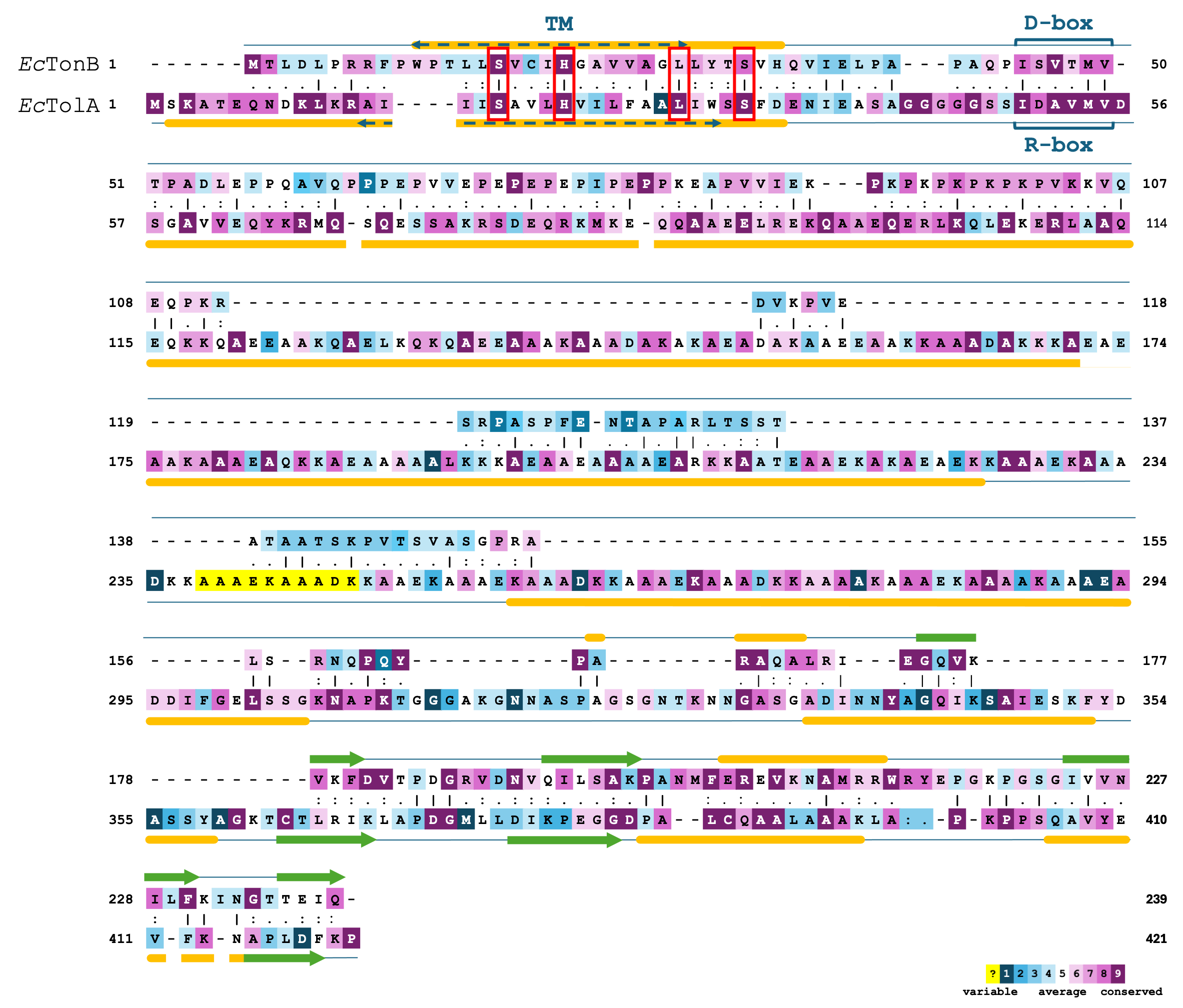
**

**C** (continued)

**
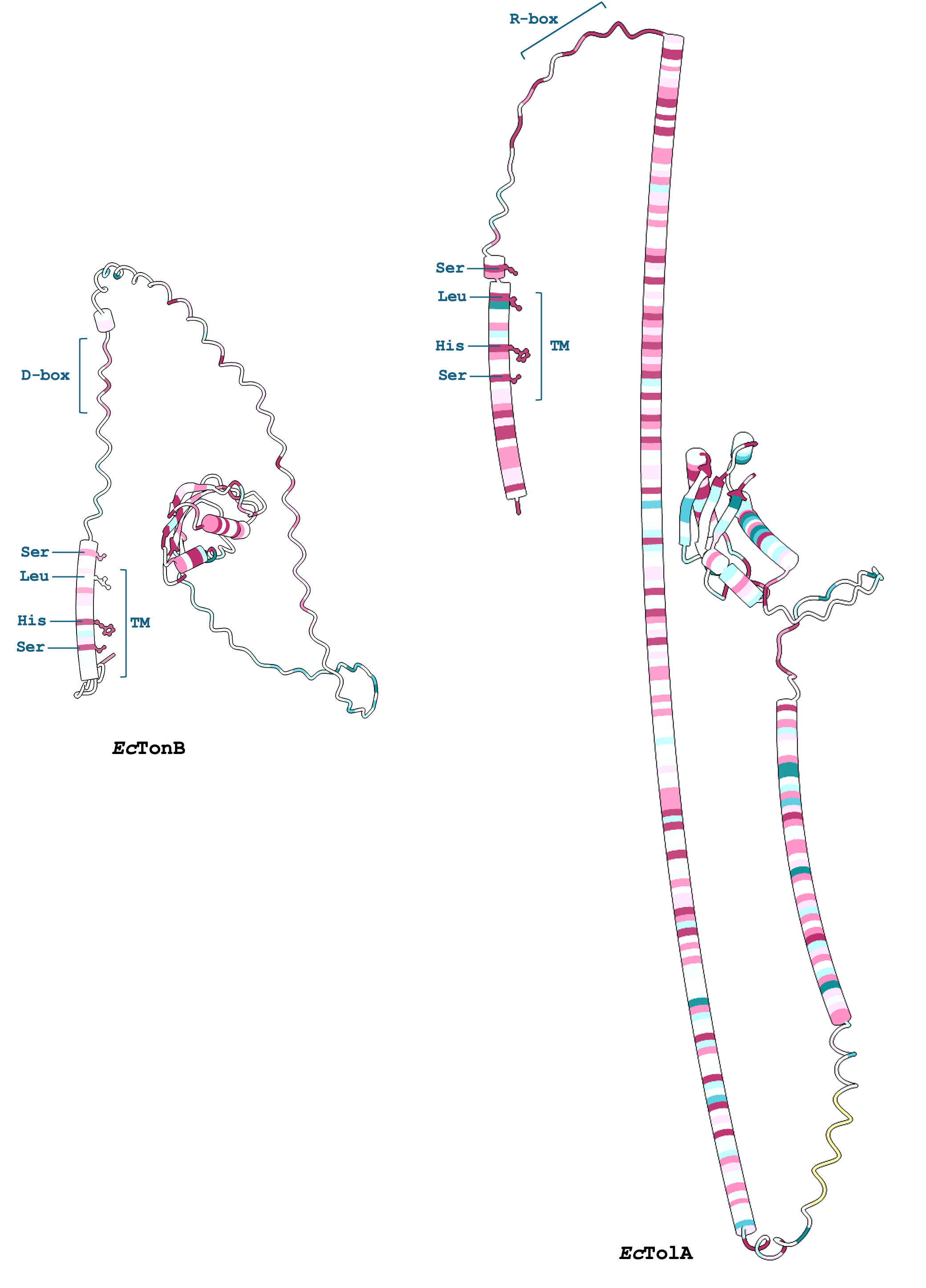
**

**Literature cited**

1. Madeira F, Madhusoodanan N, Lee J, Eusebi A, Niewielska A, Tivey ARN, et al. The EMBL-EBI Job Dispatcher sequence analysis tools framework in 2024. Nucleic Acids Res. 2024;52(W1):W521-W5. 10.1093/nar/gkae241

2. Yariv B, Yariv E, Kessel A, Masrati G, Chorin AB, Martz E, et al. Using evolutionary data to make sense of macromolecules with a "face-lifted" ConSurf. Protein Sci. 2023;32(3):e4582. 10.1002/pro.4582

3. Pettersen EF, Goddard TD, Huang CC, Meng EC, Couch GS, Croll TI, et al. UCSF ChimeraX: Structure visualization for researchers, educators, and developers. Protein Sci. 2021;30(1):70-82. 10.1002/pro.3943
